# Mapping Phosphorus Availability in Soil at a Large Scale and High Resolution Using Novel Diffusive Gradients in Thin Films Designed for X-ray Fluorescence Microscopy

**DOI:** 10.1021/acs.est.3c06237

**Published:** 2023-12-18

**Authors:** Claudia Moens, Enzo Lombi, Daryl L. Howard, Stefan Wagner, Justin L. Payne, Peter M. Kopittke, Casey L. Doolette

**Affiliations:** † Future Industries Institute, University of South Australia, Mawson Lakes, South Australia 5095, Australia; ‡ Division of Soil and Water Management, KU Leuven, Kasteelpark Arenberg 20, 3001 Heverlee, Belgium; § Australian Synchrotron, ANSTO Clayton, Victoria 3168, Australia; ∥ Department General, Analytical and Physical Chemistry, Chair of General and Analytical Chemistry, Montanuniversität Leoben, 8700 Leoben, Austria; ⊥ UniSA STEM, University of South Australia, Mawson Lakes, South Australia 5095, Australia; # School of Agriculture and Food Sciences, The University of Queensland, St Lucia, Queensland 4072, Australia

**Keywords:** DGT, X-ray fluorescence microscopy, laser-ablation ICP-MS, phosphorus fertilizer, fertosphere, spatially resolved available concentrations

## Abstract

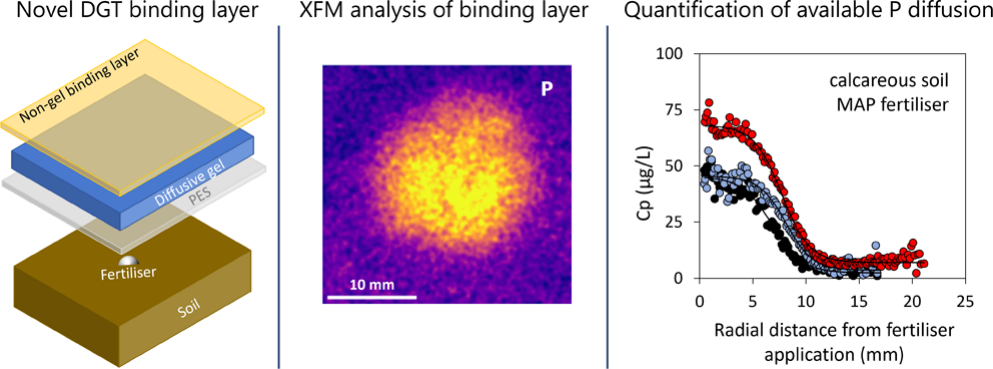

A novel binding layer (BL) as part of the diffusive gradients in thin films (DGT) technique was developed for the two-dimensional visualization and quantification of labile phosphorus (P) in soils. This BL was designed for P detection by synchrotron-based X-ray fluorescence microscopy (XFM). It differs from the conventional DGT BL as the hydrogel is eliminated to overcome the issue that the fluorescent X-rays of P are detected mainly from shallow sample depths. Instead, the novel design is based on a polyimide film (Kapton) onto which finely powdered titanium dioxide-based P binding agent (Metsorb) was applied, resulting in superficial P binding only. The BL was successfully used for quantitative visualization of P diffusion from three conventional P fertilizers applied to two soils. On a selection of samples, XFM analysis was confirmed by quantitative laser-ablation inductively coupled plasma mass spectrometry (LA-ICP-MS). The XFM method detected significant differences in labile P concentrations and P diffusion zone radii with the P fertilizer incubation, which were explained by soil and fertilizer properties. This development paves the way for fast XFM analysis of P on large DGT BLs to investigate in situ diffusion of labile P from fertilizers and to visualize large-scale P cycling processes at high spatial resolution.

## Abstract 2

A novel, easy-to-prepare, and robust binding layer for DGT and XFM analysis was developed to map phosphorus availability in the fertosphere, and significant differences based on soil and fertilizer characteristics were revealed.

## 1 Introduction

Agriculture heavily relies on phosphorus (P) fertilizers to sustain crop production as it is one of the most common nutrient deficiencies affecting crops. P adsorption and precipitation reactions in soils may reduce the mobility and availability of fertilizer applied P. The efficiency and long-term fate of fertilizer P depend both on soil properties and on the fertilizer formulation.^1,2^ To enhance fertilizer efficiency, it is essential to understand how P availability is affected by soil characteristics, fertilizer formulations and fertilization strategies. The diffusive gradients in thin films (DGT) technique^3^ is commonly used to estimate the potentially bioavailable concentrations and distribution of nutrients and contaminants in the environment. This method correlates strongly with plant available nutrients because it mimics plant nutrient uptake by acting as an infinite sink.^4^ The DGT device consists of three layers: a filter membrane that is placed in contact with the sampling surface, a diffusive gel, and a binding layer (BL) containing the analyte-specific binding agent, which immobilizes the analyte.^3^ The binding gel induces a diffusive flux toward the binding gel. After DGT deployment, the mass of the analyte accumulated on the binding gel is measured and a time-averaged flux of the analyte for the deployment time can be calculated.^3^

Visualization techniques have been developed to obtain two-dimensional (2D) images of labile P concentrations or P fluxes in soil from the DGT binding gel. These visualization methods are based on colorimetry,^5−7^ which has good detection limits but limited lateral resolution.^8^ Alternatively, X-ray fluorescence microscopy (XFM, both benchtop and synchrotron-based) and laser-ablation inductively coupled plasma mass spectrometry (LA-ICP-MS) have been used for multi-elemental mapping at high spatial resolution and low concentrations (other techniques are potentially available, e.g., NanoSIMS). Santner et al.^9^ developed the DGT LA-ICP-MS imaging technique for application in soils and made the first 2D image of mobile P in the rhizosphere. Since the first high-resolution mapping of P in soils,^9^ both laser-ablation systems^10^ and ICP-MS instruments^11^ have become much faster and able to process much higher throughput. Nevertheless, these new-generation instruments are not yet widely available, and analysis with standard LA-ICP-MS equipment is still relatively time-consuming and resource intensive, leaving LA-ICP-MS analysis mostly suited for mapping small- to moderate-sized sample areas (e.g., <25 cm^2^) at fine resolution for which high sensitivity is required.

To visualize P diffusion of fertilizers in soils or rhizosphere processes, relatively large DGTs might be required. Synchrotron-based XFM is nondestructive, which implies that a BL can be analyzed more than once.^8^ In addition, it was demonstrated recently, and for the first time, that tandem analyses of DGT can be done together with other experiments at synchrotron XFM beamlines.^12^ This means that the DGT analysis can run in the background without decreasing the beamline throughput. The study of Doolette et al.^12^ was the first using synchrotron-XFM on DGT to visualize the diffusion of labile Zn and P away from Zn–P fertilizer granules. However, it was not possible to detect P from the gel, likely because the P fluorescence signal, due to its low energy (Kα 2.014 keV), derives from shallower parts of the sample compared to the Zn fluorescence (Kα 8.637 keV). Since the P binding agent is embedded in the DGT binding gel matrix, self-absorption may have also occurred^12^ in the polyurethane-based gel, which has an approximate thickness of 100 μm.^13^ This prompted the need to design a new DGT capable of measuring P by XFM. Although designed for P detection by XFM, other light elements, which have low fluorescence energies and other surface-sensitive analyses (including LA-ICP-MS, NanoSIMS), may also benefit from a BL without gel matrix. During DGT deployment in soil, elements are present in various chemical forms including complex species, which, depending on their dissociation rates, could bind at different depths into the gel matrix.^14^ The variable binding depth between fully dissolved and complexed species might be an issue for surface-sensitive analysis in general, for which the sampling depth is limited and does not extend through the whole thickness of the binding gels.^8,15^

The aim of this study was to develop and test a new DGT BL that allows mapping the spatial distribution of labile P by the DGT technique using XFM. It was hypothesized that eliminating the gel matrix from the DGT BL might substantially increase the detectable P signal. Therefore, a new BL is proposed that has the P binding agent only at the surface of the BL. The new BL is developed first, and the quantitative XFM detection is validated by LA-ICP-MS on a subset of samples. Finally, the new BL is used to evaluate P diffusion from different fertilizers in soils with contrasting properties that are expected to show different P diffusion behavior.

## 2 Materials and Methods

### 2.1 Preparation of DGTs

In this study, a novel non-hydrogel based BL is developed and compared with a polyurethane (PU) BL, the current best BL for XFM analysis of large DGTs.^12^ The conventional DGT preparation method is given in the Supporting Information.

### 2.2 Preparation of a Novel Non-hydrogel Based BL

Currently, the DGT BL for phosphate is based on polyacrylamide with in situ precipitated zirconium (Zr).^16^ However, in XFM analysis, Zr cannot be used as a binding agent because of the overlapping fluorescence lines for the Zr L edge (Lα 2.039 keV) and P K edge (Kα 2.014 keV). Therefore, titanium dioxide-based Metsorb (MetsorbTM HMRP5, Graver Technologies) was chosen as the anionic binding agent (5 μm particle size). Metsorb is an anionic binding agent that consists primarily of titanium dioxide (TiO_2_) with smaller amounts of Ti-hydroxide (<30%) and an undescribed polymer (<10%). Metsorb was originally incorporated in DGTs by Panther et al.^17^ The novel BL for DGT analysis consists of a Kapton polyimide film backing with silicone-free acrylic adhesive on one side (12″ × 12″ 3 M Low Static Polyimide Film Tape 7419, product number 12X12-6-7419 purchased through DigiKey (digikey.com.au)) to which the Metsorb was applied homogeneously using a makeup brush (Natio Bronze & Highlight Brush). An acrylic adhesive was used instead of the more common Si-based adhesive to avoid interference from what would have been a strong Si emission signal (Kα 1.739 keV). Next, the Ti-loaded BL is placed on a clean weighing paper with the Metsorb facing the paper and pressed to firmly attach the Metsorb to the film. Finally, any Metsorb not attached to the adhesive is removed by gently brushing the surface. Gently streaming compressed air across the tape was initially considered for removing any unbound Metsorb, but preliminary experiments showed no improvement in Metsorb adherence (determined by weighing the Metsorb mass on the tape before and after air blowing), so this step was therefore omitted. The Ti content of the BL was determined after microwave digestion^18^ of six BLs (7.5 cm^2^) using ultrapure 70% HNO_3_ (2 mL) with 98% H_2_SO_4_ (4 mL) to dissolve TiO_2_ (45 min of digestion at 210 °C with the Mars 6 microwave system, CEM Corporation), with the Ti content analyzed using ICP-MS (Agilent 8900). The digestion method resulted in complete dissolution of the Kapton tape. The distribution of Ti on the BL was determined by mapping Ti by XFM analyses (see below).

### 2.3 Soil-Fertilizer Incubation Experiment

#### 2.3.1 Soil Properties

Two contrasting Australian soils were selected to investigate the diffusion of various P fertilizers in soils. The first is a Calcarosol (Australian Soil Classification) clay loam from South Australia (denoted as “SA soil”), and the second soil is a clay Chromosol (Australian Soil Classification) from New South Wales (denoted as “NSW soil”). These two soils are, respectively, classified as Calcisols and Luvisols in the World Reference Base. The soils were air-dried, sieved to ≤2 mm, and stored dry. Selected soil properties (Table S1) have previously been determined.^5^

#### 2.3.2 Fertilizer Incubation Experiment

A fertilizer-soil incubation experiment was set up in round Petri dishes (90 mm in diameter, 15 mm in height) as previously described by Lombi et al.^2,19^ Briefly, air-dried and sieved (<2 mm) soil was prewetted to 50% of the water holding capacity (WHC) using deionized water 1 day before filling the Petri dishes. The premoistened soil, equivalent to 75 g dried soil, was added to Petri dishes, and the moisture content was increased to 80% of the WHC in the Petri dishes. The Petri dishes were sealed with parafilm and incubated at 25 °C for 7 days. Next, three different P fertilizers were added to the incubated soils. Phosphorus was applied at the center of each Petri dish in one of three commercial fertilizer formulations, two granular P fertilizers (1) monoammonium phosphate (MAP) (Incitec Pivot Fertilisers) and (2) diammonium phosphate (DAP) (Incitec Pivot Fertilisers), and one liquid P fertilizer ammonium polyphosphate (APP, Polyphos by Agrichem), all at the same P rate of 10.0 mg P per dish. Thus, the experiment consisted of two soils and three different P fertilizers, with each treatment being replicated three times, leading to 18 Petri dishes in total for the P diffusion experiment with the Kapton BL. Three additional Petri dishes with SA soil and MAP fertilizer were included to test the effect of DGT application without the diffusive layer (DL). In addition, two additional Petri dishes with both soils and MAP fertilizer were set up to test the PU BL. The P content of the fertilizers was verified by ICP-MS following microwave-assisted acid digestion (30 min at 180 °C) using 8 mL of concentrated (70%) HNO_3_ (100 ± 5 mg sample). MAP and DAP fertilizer granules were weighed, and single granules corresponding to 10.0 mg P were placed in the center of the Petri dish and then pushed down (5 mm) to half the soil depth using a pin. For the liquid APP, 50 μL was added with a needle syringe for placing this fertilizer in the same position. To compensate for the liquid added with APP, 50 μL of demineralized water was added together with the MAP and DAP. After fertilizer addition, the Petri dishes were sealed with parafilm again and incubated for 35 days at 25 °C.

### 2.4 DGT Deployment in Soils Amended with P Fertilizer

For soil deployment, the novel Kapton BL and PU BL were assembled as conventional DGT methods, i.e., the poly(ether sulfone) (PES) membrane and bis-acrylamide diffusive gel were placed between the soil surface and the BL. One additional treatment was included (SA soil with MAP fertilizer) where the diffusive gel was omitted to assess whether lateral diffusion in the diffusive gel caused image blurring.^20^ The size of the diffusive gel and BL was 50 × 50 mm square. A 3D printed support (Vero Clear resin) was made with a Polyjet J735 printer to apply the DGT exactly in the center of the Petri dish and to ensure uniform contact of the DGT with the soil. The soil was additionally moistened right before DGT deployment to ensure good soil-gel contact, and the Petri dish was closed and sealed with parafilm with the lid of the Petri dish placed on top of the gel layers to obtain a good connection between the soil and the DGT layers. The DGT was deployed for 72 h in a temperature-controlled room at 20 °C to obtain an optimum P loading for subsequent XFM analyses. The deployment time was determined from a preliminary experiment in which the deployment time was varied (24, 48, and 72 h), and P was analyzed by benchtop XFM (results not shown). After deployment, the membrane and DL covering the BLs were removed, and the Kapton BL was allowed to quickly dry in the plastic support. The PU BLs were transferred, the reactive side facing upward, onto a wetted 0.45 μm cellulose acetate membrane (Supor 450, Pall Life Sciences) to minimize shrinkage and dried at room temperature for 2 days. The gels and underlying 0.45 μm membrane were inseparable from each other after drying. All BLs were analyzed by synchrotron-based XFM (below), and a small selection of the Kapton BLs was subsequently analyzed by LA-ICP-MS.

### 2.5 Spatial Distribution of Labile P

#### 2.5.1 XFM Imaging

The X-ray fluorescence mapping was performed at the XFM beamline of the Australian Synchrotron (ANSTO) in Melbourne, Victoria.^21^ Samples are analyzed at the microprobe end-station with a Vortex EM fluorescence detector, which allows to detect photons with energy above approximately 1.6 keV. The DGT BLs were mounted on Perspex sample mounts using double-sided tape. The aperture width of the sample mount is approximately 40 mm; the full width of the BLs (50 mm) could not be scanned due to scattering from the edges of the frame. All DGTs were analyzed twice: first a quick coarse scan (resolution 200 μm) to locate the region of interest, followed by a fine resolution scan (resolution 30–50 μm) to collect the desired image. Due to limited P diffusion in the SA soil, smaller areas were analyzed than in the NSW soil. The samples were scanned with the horizontal axis in continuous motion but with discrete vertical steps that matched the resolution in the *x*-direction (200, 50, or 30 μm). The transit time per pixel was set to 16.6 ms. The photon energy of the incident X-ray beam was set at 4.8 keV using a Si(111) monochromator. To improve sensitivity, a continuous flow of helium (at a flow rate of 200 mL/min) was applied through the collimator of the Vortex to reduce Ar fluorescence and reduce sorption in air. To allow for quantitative analysis, the detector response was calibrated by measuring metal foil standards at the start of the beamtime. The P fluorescence spectra were analyzed, and elemental P loadings on the BL were quantified and visualized using GeoPIXE.^21,22^ The P mass loadings (ppm) on the BLs calculated with GeoPIXE were exported to Microsoft Excel and converted to 2D maps of P surface loadings (ng cm^–2^) using matrix-matched DGT calibration standards with different P loadings (see below). The DGT standards were additionally analyzed for Ti with the energy of the incident X-ray beam set at 10.0 keV to visualize the homogeneity of the Metsorb distribution on/in the BLs.

#### 2.5.2 Calibration Standards

Matrix-matched DGT standards for calibration of the XFM and LA-ICP-MS signal intensities were prepared by exposing Kapton BLs (*A* = 7.5 cm^2^) in duplicates to solutions with six different P concentrations on an orbital shaker at pH 6.5 and 0.01 mol L^–1^ NaCl background. The BLs were exposed for 24 h, thereby yielding six different P loadings, which were determined by XFM and, after elution, by ICP-MS as described in the Supporting Information.

#### 2.5.3 LA-ICP-MS Imaging

A 193 nm ArF excimer-based Iridia laser-ablation system (Teledyne-CETAC) was coupled to triple-quadrupole ICP-MS (8900 Agilent) for the analysis of a selection of DGT BLs and matrix-matched standards. The settings of the LA-ICP-MS setup are given in Table S2. The spot size was set at 80 μm and scanned at 1600 μm s^–1^ with an ICP-MS duty cycle of 100 ms, resulting in pixel sizes in the scan direction of 160 μm. Horizontal lines were ablated on the gels with vertical spacing of 160 μm to obtain square 160 × 160 μm pixels. The total analysis time per gel ranged between 180 and 200 min (excluding calibrations). The measured mass-to-charge ratios (*m*/*z*) were 31 (P), 13 (C), and 47 (Ti). Internal normalization by C or Ti did not improve the image analysis, and therefore, the analysis of these elements is not used for further evaluation. Elemental surface loadings (ng P cm^–2^) were determined using the calibration function derived from matrix-matched DGT standards. The processing of the LA-ICP-MS data is described in the Supporting Information.

### 2.6 Data Analysis

The 2D maps of surface loadings of P on the BL (*M*_P_ in ng P cm^–2^) obtained with XFM analysis were converted to 2D maps of labile P concentrations (*C*_P_ in μg P L^–1^) using the DGT equation (eq 1)^3^
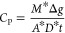
1where *M*/*A* (ng P cm^–2^) is the P surface loading on the BL accumulated during the deployment time, Δ*g* is the DL thickness (cm), *D* is the diffusion coefficient for PO_4_ through a membrane-based bis-acrylamide DL,^5^ and *t* is the deployment time (s). The diffusion coefficients of polyphosphates (in the APP fertilizer) in the DL were not determined, but it is expected that they would be slightly lower than that of PO_4_ (see Section 6 of the Supporting Information).

The 2D maps of *C*_P_ are presented as contour plots plotted using the software program R v4.2.3^23^ for which *C*_P_ values <0 are set at zero. To quantitatively compare P diffusion in both soils and different fertilizers, the *C*_P_ concentrations were plotted as a function of the radial distance (*R*) from the fertilizer application using ImageJ (see Supporting Information for details). The P diffusion profiles measured in the samples generally exhibited a sigmoidal pattern. Therefore, a four-parameter log-logistic model describing the *C*_P_ concentration as a function of radial distance (eq 2) was fit on each of the plots, and relevant model parameters were statistically compared.
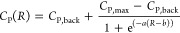
2where *C*_p_(*R*) is the *C*_P_ (μg P L^–1^) concentration as a function of radial distance *R* (mm) from the fertilizer application, *C*_P,back_ (μg P L^–1^) is the lower asymptote value corresponding to the *C*_p_ concentration far from fertilizer application, *C*_P,max_ (μg P L^–1^) is the upper asymptote value corresponding to the concentration at fertilizer application, and *a* (mm^–1^) and *b* (mm) parameters of the log-logistic model related to the slope and point of inflection. The profiles of all curves were fitted with the nonlinear fitting routine with the statistical software JMP (JMP, Version 17. SAS Institute Inc., Cary, NC, 1989–2023).

The radius of P diffusion (*R*_diff_) is defined here as the distance from fertilizer application where *C*_P_ decreases below the limit of quantification (LOQ) (10 times the standard deviation in blanks, 24 μg of P L^–1^) determined from the XFM calibration curve. The differences in parameter values *C*_P,max_ and *R*_diff_ among different fertilizers of the same soil or among soils are compared statistically using the student *t* test at significance level α < 0.05 using individual Petri dishes as sampling replicates (three). To compare the XFM and LA-ICP-MS analyses, a similar data analysis was done on the P surface loadings (*M*_P_) on the BL measured with both methods, which is described in more detail in the Supporting Information.

## 3 Results and Discussion

### 3.1 Need for a New DGT BL

Previously, Doolette et al.^12^ successfully used synchrotron-based XFM analysis of DGT gels (PU-based) to visualize the diffusion of labile Zn away from Zn–P fertilizer granules. However, no P fluorescence signal could be detected from the gel-based DGT BL, which was explained by absorption of the P fluorescence signal in the polyurethane gel matrix, since the P binding agent is embedded in that gel. Indeed, theoretical calculations considering PU properties, density, and thickness, support this explanation of high fluorescence absorption for P—a PU gel layer that is only 10 μm in thickness would already reduce the P fluorescence signal (2.1 keV energy) by almost 40%, while >80% is absorbed by a 50 μm thick PU layer (Figure S1). This is in contrast with the heavier element Zn (8.637 keV), for which even a 100 μm thick PU gel only absorbs 4.1% of the fluorescence signal confirming the previous observations.^12^ To overcome these issues with P detection, we proposed two methods. First, improve P detection using a more sensitive detector for P (these previous analyses were conducted using the Maia fluorescence detector system, see below); second, develop a new non-hydrogel based DGT BL to increase the P fluorescence signal by eliminating the gel matrix in which Metsorb is embedded. The Metsorb-based PU gels were prepared to verify if P detection from these gels can be improved using the Vortex detector instead of the previously used Maia detector system. The Vortex EM fluorescence detector allows to detect photons with energy above approximately 1.6 keV in contrast to the lower-energy detection cutoff of approximately 2.0 keV of the Maia detector. To maximize the sensitivity, a flow of helium was also applied through the collimator of the Vortex to reduce air absorption losses.

The results of the PU gel analysis showed that P detection was improved using the Vortex detector, with P fluorescence detected on the gels, whereas this was not detected previously with the Maia system (Figure S2, panels A and B). However, the P distribution was not as expected based on the experimental setup with the P fertilizer in the center of the DGT. Analyzing two treatment replicates also showed a very different P distribution on both gels. These results could be caused by a heterogeneous distribution of the Metsorb through the thickness of the gel matrix, which would distort the apparent P distribution. This is in contrast to the P fluorescence detection from two Kapton-based BLs, which showed a P distribution profile as expected, i.e., a P diffusion profile that is maximal at the center of the BL where the P fertilizer is applied (Figure S2, panels C and D). Hence, despite the improved P fluorescence detection (higher signal) with the Vortex detector, the process of self-absorption still confounded the measurements, leading to the unrealistic P distribution on the PU gel. In contrast, the P detection from the Kapton-based BL was promising and is therefore further discussed below.

### 3.2 Characteristics of the New Kapton-Based BL

Preparation of the Kapton BL is straightforward and quick. This contrasts with conventional gel-based methods that require several preparation steps before use.^24^ In addition, handling of the Kapton BLs is also very convenient as it does not tear and can easily be cut to the desired size and shape. Finally, after deployment, the BLs quickly air-dry and do not shrink, which is an important advantage for high-resolution imaging and can be an issue with current gel-based BLs.^12^ The Ti content of the BL was 29 ± 2 μg Ti cm^–2^ measured on six BLs of 7.5 cm^2^, with the low standard deviation indicating the homogeneous application of Metsorb on the tape (which is also visually shown in Figure S3). The Ti distribution on the PU gel and Kapton BL was also mapped with XFM (Figure S4). The Ti was more homogeneously distributed on the Kapton BL than on the PU gel given the 10-times lower standard error for the Ti signal. Of note, however, is that the average Ti signal is approximately four times lower for the Kapton BL than for the PU gel, which could result in a lower P sorption capacity for the Kapton BL than for the PU gel. Finally, the Ti concentrations in equilibrium blank and P solutions for calibration (see below) were below the ICP-MS detection limit, indicating minimal Ti release from the tape during deployment in solution. Following DGT disassembly, there were no visual indications that Metsorb was released from the Kapton tape and adhered to the DL. From the analysis of the BL, there were also no irregularities in P distribution to suggest loss of the P binding agent, Metsorb, from the BL.

### 3.3 Calibration of 2D Maps from XFM Analysis with Matrix-Matched Standards

A P calibration curve with Kapton BLs was determined following 24 h of adsorption in P solutions at pH 6.5. The calibration curve is determined by plotting the P mass loading (ppm) measured by XFM against the P mass loadings (ng P cm^–2^) on the BLs measured after elution. A linear calibration curve was obtained with a *R*^2^ of 0.99 (Figure S5). The calibration curve was used to calculate all P mass loadings from the XFM fluorescence signal. The average P loading on the blanks (*n* = 2) is slightly elevated, 46 ng P cm^–2^, but still at the low end of the P loadings in the samples, and therefore, no subtraction was made for the P loading on the blanks. The limit of detection (LOD) of P (three times the standard deviation of blanks) was 41 ng of P cm^–2^. This LOD value corresponds to a *C*_P_ concentration of 7.3 μg P L^–1^ using eq 1 with deployments for 72 h and a phosphate diffusion coefficient of 1.74 × 10^–6^ cm^2^ s^–1^. As is always the case with XFM, the detection can be improved by increasing the transit time per pixel (although this also increases the scanning time). With the settings used here, the LOD is slightly below the LOD of a recently developed colorimetric DGT method for P imaging,^5^ i.e., 50 ng P cm^–2^. The maximum P loading in the calibration experiment is approximately 360 ng P cm^–2^, but future work is needed to characterize the maximum linear accumulation capacity of P by this new BL.

### 3.4 Comparison of P Diffusion between XFM and LA-ICP-MS

**Figure 1:**
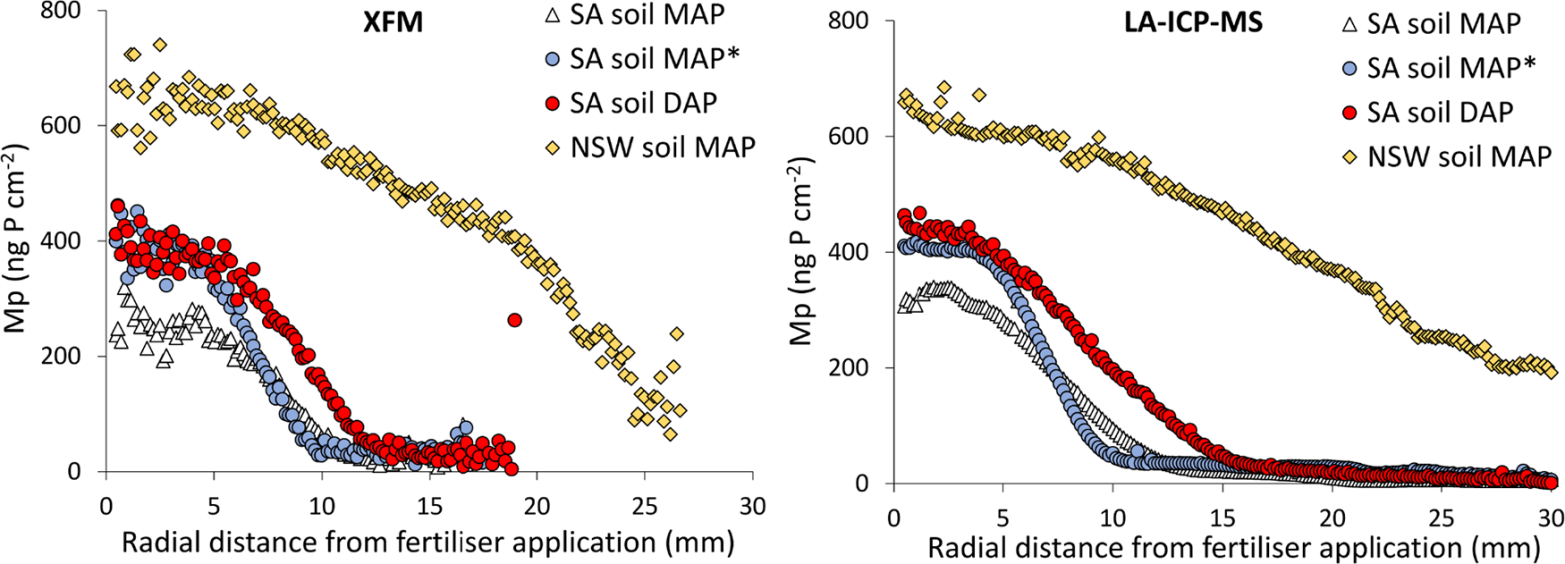
XFM (left) and LA-ICP-MS (right) analysis of the P loading *M*_p_ (ng P cm^–2^) on Kapton BLs as a function of radial distance of fertilizer application in the South Australian (SA) soil and New South Wales (NSW) soil with MAP and DAP fertilizers. MAP* indicates that the DGT was deployed without a diffusive layer.

Although the Iridia short pulse module from the cobalt cell allows rapid per pixel analysis times in the range of XFM analysis (16.6 ms), the settings for LA-ICP-MS analysis (dwell time longer at 100 ms, pixel size larger) were slower than this as a trade-off between resolution, sensitivity, and analysis time. The sensitivity of P detection, derived from the calibration curve, can therefore not be directly compared between XFM and LA-ICP-MS, but the aim was to verify the quantitative XFM analysis. To validate the XFM analysis of the Kapton BLs, we analyzed selected BLs with both methods. The analyses were conducted on the same BLs consecutively, first the nondestructive XFM analysis followed by LA-ICP-MS analysis. Figure 1 shows that the P distribution on the BLs was in very good agreement between the two methods. Both methods determined a similar radius of P diffusion (*R*_diff_), with *R*_diff_ MAP* (without DL) < *R*_diff_ MAP < *R*_diff_ DAP ≪ *R*_diff_ NSW (Table 1). For both methods, the P loading at the center of fertilizer application (*M*_P,max_) increased in the order MAP SA (with and without DL) ∼ DAP SA < MAP NSW. The curve fitting results (Table 1) indicate that P loadings (*M*_P,max_) are 5–32% higher when assessed with LA-ICP-MS than with XFM. The LA-ICP-MS analysis showed a large decrease in sensitivity during the analysis which was corrected for by interpolation from calibration at the start and end of the analysis (Figure S6), the largest uncertainty is therefore at intermediate analysis time, which corresponds to the center of the BL (hence *M*_P,max_). It is noted here that future LA-ICP-MS analysis can be improved with additional calibration or check samples during the analysis of a BL to better correct for the large drift in sensitivity (Figure S6). The widths of the diffusion profiles obtained with or without a diffusive gel were equal, thereby indicating that lateral diffusion of P within the DL did not occur (Table 1 and Figure S7). Therefore, a diffusive gel was used in all experiments to obtain P concentrations comparable to labile concentrations from conventional DGT bulk samplers^20^ (further discussed in Section 13 of the Supporting Information).

**Table 1:** Comparison of XFM and LA-ICP-MS Analyses on a Selection of Kapton BLs Measuring the P Diffusion Profiles in Two Soils with MAP and DAP Fertilizers^a^

	XFM analysis		LA-ICP-MS analysis	
	*M*_P,max_, ng P cm^–2^	*R*_diff_, mm	*M*_P,max_, ng P cm^–2^	*R*_diff_, mm
South Australian soil				
MAP	257	9.6	341	9.8
MAP^b^	397	9.0	415	8.6
DAP	391	11.7	473	12.8
New South Wales soil				
MAP	702	28.1	812	32.4

a *M*_P,max_ is the P loading on the Kapton BL at the fertilizer application zone and *R*_diff_ is the radius of P diffusion, derived from the plots in Figure 1.

b DGT deployed without a diffusive layer.

### 3.5 Comparison of P Diffusion in Two Contrasting Soils

**Figure 2:**
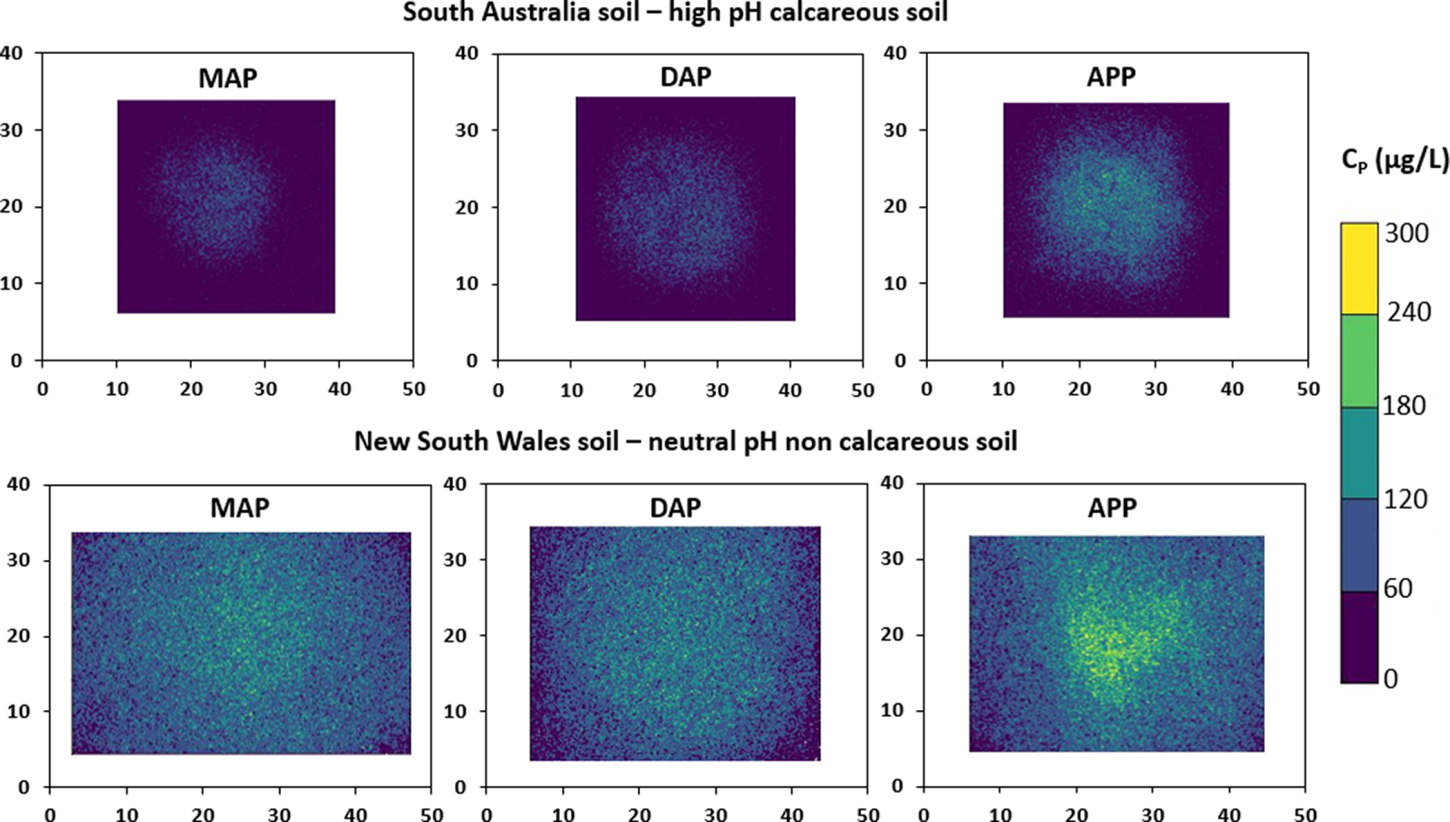
2D diffusion profiles measured with XFM on Kapton BLs. The top shows the limited diffusion in the strongly P fixing soil of South Australia, and the bottom shows the more extensive P diffusion in the New South Wales soil, both for three different P fertilizers (MAP, DAP, and APP). The axes indicate the scale (mm).

**Figure 3:**
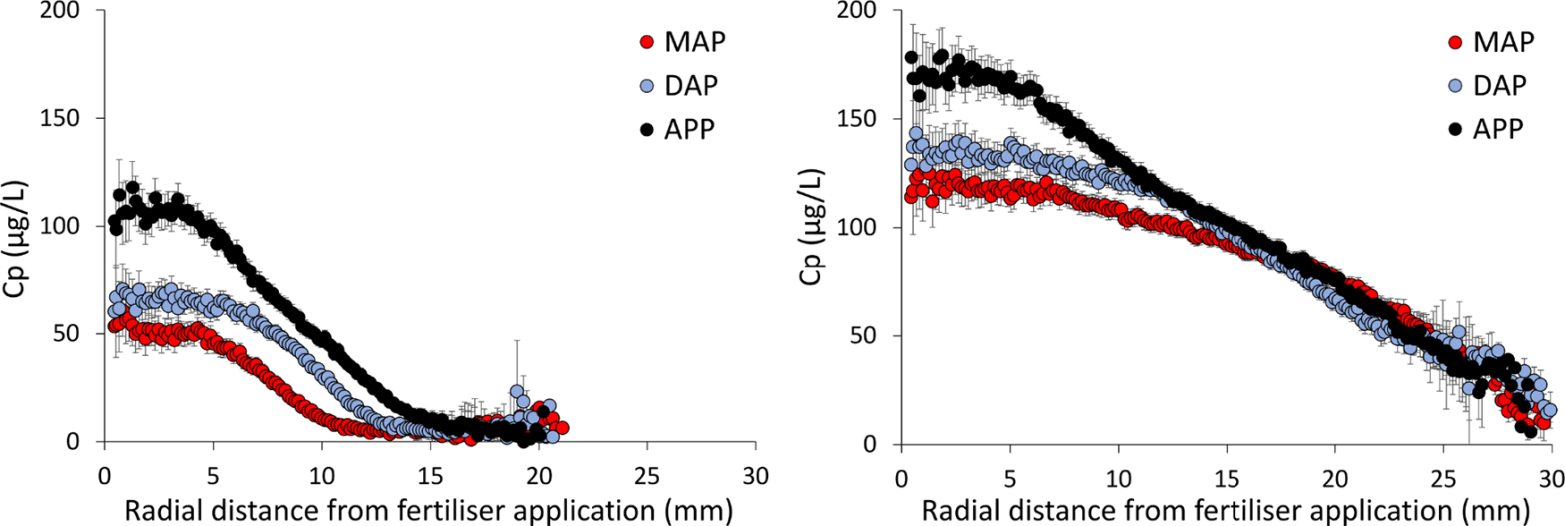
XFM analysis showing the C_p_ concentration (μg P L^–1^) as a function of radial distance of fertilizer application in the South Australian soil (left) and New South Wales soil (right) with MAP, DAP, and APP fertilizers derived from the 2D diffusion profiles on Kapton BLs. The points are means of triplicate analyses, and the bars are their 95% confidence intervals.

In soil, P diffusion depends on soil physical factors, including bulk density, water content, texture (i.e., tortuosity), and sorption/precipitation reactions, that are largely controlled by soil pH, presence of CaCO_3_, and the concentration of Al and Fe oxides. At lower pH, amorphous Al and Fe oxides are the main sorbent of P in soils and P diffusion decreases with increasing oxalate-extractable Al and Fe^1^ or precipitation of aluminum-P minerals occurs.^25^ In soils with higher pH values, P diffusion can be limited due to sorption on CaCO_3_ and the formation of Ca–P precipitates.^1^ The two soils used here are the alkaline, calcareous SA soil ( 8.5), which has a high capacity for binding P, and the more neutral pH clay NSW soil ( 7.4), which is more conducive to P diffusion concluded from the phosphorus buffering index (PBI), which is moderately high in the SA (180) and low in the NSW soil (73) (Table S1). Both soils also had a low initial P status (low Colwell and Olsen P, Table S1). Figures 2 and 3 show two main features regarding P diffusion in these soils: (1) P diffusion was much more pronounced in the NSW soil than in the SA soil regardless of the fertilizer type and (2) the difference in P diffusion between fertilizers is larger and significant in the SA soil compared to the NSW soil.

Phosphorus concentrations in the 2D images (C_P_) ranged from ≤7.3 μg P L^–1^ (blue, LOD) to ≥200 μg P L^–1^ (yellow) with strong gradients in P concentrations decreasing from the point of fertilizer application (Figure 2). The P was relatively immobile in the SA soil, evidenced by the radius of P diffusion (*R*_diff_) which was limited to 7.9–12.3 mm from the fertilizer application point, with P concentrations (*C*_P,max_) ranging from 53 to 115 μg P L^–1^ at the center of fertilizer application. In the NSW soil, the radius of P diffusion was approximately 3-fold larger, up to 30.6–33.1 mm, with the P concentration at the fertilizer application up to 124–195 μg P L^–1^. These results are therefore in agreement with those of the PBI and suggest that the P fixation reactions largely control diffusional differences between both soils. Arias et al.^5^ previously measured P diffusion from MAP granules in the same soils using a colorimetric DGT approach. The trends in P diffusion were the same, i.e., a much larger P diffusion in the NSW soil than in the SA soil, albeit the diffusion gradient was much less clearly defined in the study of Arias et al.^5^ The low P diffusion was explained by the likely precipitation of calcium phosphates around the fertilizer granule given that the solubility product of phosphate minerals is locally exceeded; when P moves away from the granule and the P concentration in soil solution decreases, adsorption becomes the dominant process.^5^ In the relatively neutral NSW soil, precipitation and adsorption reactions were less prevalent because of the low CaCO_3_ content and low Al concentration.^5^ The P concentrations around the granule obtained by Arias et al.^5^ (<7 mm, approximately 250 μg P L^–1^ NSW and approximately 150 μg P L^–1^ SA) agreed reasonably well with the C_P_ concentrations determined here (e.g., *C*_P,max_ 124 ± 18 μg P L^–1^ NSW and 53 ± 13 μg P L^–1^ SA) despite the large differences in experimental setup between both studies (e.g., colorimetric against fluorescence detection and different DGT BLs).

### 3.6 Comparison of P Diffusion in Soil from Different P Fertilizers

In the calcareous SA soil, statistical differences are found in *C*_P,max_ in the order APP > MAP = DAP and in the radius of P diffusion *R*_diff_ in the order APP > DAP > MAP (Table 2). In the NSW soil, *C*_P,max_ is significantly higher for APP compared to the equal *C*_P,max_ for MAP and DAP, but no significant differences were observed among the different fertilizers in terms of *R*_diff_, where APP = DAP = MAP (Table 2). The P diffusion profiles for all individual sample replicates together with the log-logistic model fit are given in Figure S8.

**Table 2:** Comparison of P Diffusion Profiles in Two Soils That Received MAP, DAP, and APP Fertilizers^a^

	XFM analysis			
	*C*_P,max_, μg P L^–1^		*R*_diff_, mm	
South Australian soil				
MAP	53 ± 13	B	7.9 ± 1.1	C
DAP	66 ± 5	B	10.5 ± 0.4	B
APP	115 ± 10	A	12.3 ± 0.8	A
New South Wales soil				
MAP	124 ± 18	B	31.1 ± 3.7	A
DAP	138 ± 15	B	33.1 ± 4.7	A
APP	195 ± 14	A	30.6 ± 5.5	A

a *C*_P,max_ is the concentration at the fertilizer application zone, and *R*_diff_ is the radius of P diffusion (average ± standard deviation). The parameters are derived from the plots in Figure 3. The letters indicate significant differences (*p* < 0.05) between *C*_P,max_ and *R*_diff_ among the fertilizers for both soils.

The saturated solutions of MAP and DAP fertilizers have markedly differing pH values; the saturated solution of DAP is alkaline (pH 8) whereas that of MAP is strongly acidic (pH 3.5).^26^ Previous fertilizer-soil incubation experiments have shown contrasting results in terms of P availability and diffusion with the application of MAP and DAP at the same P rate in the same soil. Higher P solution concentrations and/or more diffusion with MAP than DAP has been observed and is explained by lower pH around MAP resulting in less precipitation of Ca phosphate minerals. For instance, in a Vertisol, Meyer et al.^25^ observed higher initial solution P concentrations with MAP compared to DAP due to the pH decrease around MAP bands with dissolution of pre-existing Ca–P or primary P minerals. Likewise, in a high-pH soil with low CaCO_3_ content, Degryse and McLaughlin^1^ observed more diffusion with MAP than DAP likely due to more precipitation of Ca phosphate minerals with DAP as the P source. Conversely, in soils where Fe and Al dominate the P mobility, there is usually more P diffusion from DAP compared with MAP. In such soils, the increase in pH following DAP dissolution decreases P sorption to Fe and Al oxides^1^ and reduces precipitation of P and Al cations in solution at the higher pH.^25^ In our SA soil, which has a high pH and is highly calcareous, more P diffusion occurred with DAP as the P source than MAP. We hypothesize that in a soil where the pH is highly buffered by CaCO_3_, the local acidification of the MAP fertosphere causes increased dissolution of carbonates and increased Ca^2+^ activity, which can then react with the phosphate ions to produce poorly soluble Ca phosphates. In the NSW soil, no difference was observed between MAP and DAP and, P behavior appeared to be unrelated to the form of P fertilizer used as previously observed for soils with low P retention capacity.^2^ The significantly higher *C*_P,max_ of the APP fertilizer in the NSW soil compared to those of MAP and DAP is therefore somewhat surprising. The fertilizer treatment with APP was the best performing in terms of radius of P diffusion and availability in both soils, with significantly higher *C*_P,max_ and *R*_diff_ in the SA soil and significantly higher *C*_P,max_ in the NSW soil compared with the other P fertilizers.

The APP treatment contrasts with the other fertilizer treatments in formulation (liquid versus granular fertilizer) and in P speciation (polyphosphate versus orthophosphate). It was anticipated that the liquid formulation would maximize the diffusion of P from the site of application, especially in the calcareous SA soil. It has been shown that P derived from a range of fluid fertilizer products is consistently more mobile, soluble, and labile than P applied as granular forms in highly calcareous soils, but very little difference in alkaline noncalcareous soils.^2,19^ At the end of the incubation experiment after DGT application, MAP and DAP granule residues were still found in both soils, which indicates that water-insoluble compounds present in granular products did not dissolve completely in the high pH environment of these soils. The good performance of the APP is likely controlled by its distinct P speciation. The APP was previously shown to behave slightly better than other fluid fertilizer forms.^2^ Ammonium polyphosphate contains orthophosphate, pyrophosphate, and, to a lesser extent, tripolyphosphate and more condensed P forms. The P speciation changes due to hydrolysis reactions where more condensed P species react with water to form less condensed forms of P.^27,28^ The hydrolysis of polyphosphates is driven by microbial activity or by chemical hydrolysis at low pH. Polyphosphates may prevent or delay precipitation reactions through the formation of soluble complexes with dissolved cations.^29^ In addition, polyphosphates can be adsorbed onto mineral surfaces in soil like orthophosphate but can still undergo hydrolysis resulting in the release of orthophosphate over time.^30^ As the soils of this study had a neutral to alkaline pH and relatively low to moderate organic carbon content (0.8–2.1%), a low hydrolysis rate for polyphosphate is expected, resulting in high labile P concentrations in both soils.

### 3.7 Significance and Outlook

This study shows that reactions of different forms of P fertilizers in the fertosphere are complex, and this novel DGT design with a gel-free BL can provide an easy and convenient method to visualize P availability in 2D. To improve fertilizer efficiency, mechanisms that control fertilizer-soil interactions need to be better understood. The novel method described provides a way forward. Specifically, this method can be used to measure spatially resolved concentration gradients of labile P in the fertosphere and surrounding soil, which cannot be obtained using bulk soil analyses. This novel design is suitable for both XFM (both synchrotron- and laboratory-based) and LA-ICP-MS. In fact, quantitative P availability distribution on the Kapton BL measured by XFM was verified by LA-ICP-MS analysis using calibration with matrix-matched standards. As far as known, this is the first study validating DGT imaging with two independent visualization methods. Although the design of the BL was driven by the need to measure P with XFM, we believe that this new BL might be adopted more widely as an alternative BL in DGT analysis, but further research is needed to determine the binding characteristics of P by this new layer. Its main advantages are its quick and facile preparation, easy handling, robustness, and lack of shrinkage upon drying. Combined with fast XFM detection capabilities, this novel DGT design allows exploration of P availability in 2D at a significantly larger scale than what obtained thus far. This could open the door to the investigation of P dynamics under field conditions where spatial heterogeneity requires a larger scale investigation.

## Supporting Information Available

Additional experimental details, materials and methods, including soil properties, Kapton BL calibration standards, LA-ICP-MS imaging, settings for LA-ICP-MS, image processing and statistical analysis, discussion of DL thickness and figures of P fluorescence signal absorption, XFM and LA-ICP-MS calibration curves, and additional XFM images (PDF)
