# Supplementary material for: Mapping Phosphorus Availability in Soil at a Large Scale and High Resolution Using Novel Diffusive Gradients in Thin Films Designed for X-ray Fluorescence Microscopy
Source: Environ Sci Technol. 2023 Dec 18;58(1):440–8. doi: 10.1021/acs.est.3c06237 (PMC10785753; doi:10.1021/acs.est.3c06237)
Supplement: Supplementary file 1 — es3c06237_si_001.pdf [file es3c06237_si_001.pdf]

Supporting information

Mapping phosphorus availability in soil at large scale and high resolution using a novel DGT designed for X-ray fluorescence microscopy

Claudia Moens[1,2,], Enzo Lombi[1], Daryl L. Howard[3], Stefan Wagner[4], Justin L. Payne[5], Peter M. Kopittke[6] and Casey L. Doolette[1*]

1 University of South Australia, Future Industries Institute, Mawson Lakes, South Australia 5095, Australia

2 KU Leuven, Division of Soil and Water Management, Kasteelpark Arenberg 20, 3001 Heverlee, Belgium

3 Australian Synchrotron, ANSTO Clayton, Victoria, 3168, Australia

4 Montanuniversität Leoben, Department General, Analytical and Physical Chemistry, Chair of General and Analytical Chemistry, 8700 Leoben, Austria

5 University of South Australia, UniSA STEM, Mawson Lakes, South Australia 5095, Australia

6 The University of Queensland, School of Agriculture and Food Sciences, St Lucia, Queensland 4072, Australia

*Corresponding author: Casey Doolette, email: Casey.Doolette@unisa.edu.au

Contents

1. DGT preparation material and methods
2. Table S1 Soil Properties
3. Kapton binding layer calibration standards
4. Laser Ablation ICP-MS imaging
5. Table S2 Settings laser-ablation (LA) ICP-MS
6. Image processing and Statistical analysis
7. Figure S1 P fluorescence signal absorption
8. Figure S2 2D maps of polyurethane binding gels versus kapton BL
9. Figure S3 Visual distribution of Metsorb on the Kapton BL
10. Figure S4 2D map of Metsorb distribution for polyurethane binding gels and kapton BL
11. Figure S5 Calibration curve XFM
12. Figure S6 Calibration curve LA-ICP-MS
13. Diffusive layer thickness
14. Figure S7 P loading on Kapton BLs as function of radial distance with and without DL
15. Figure S8 Log-logistic model fit on individual sample replicates
16. References

1. DGT preparation material and methods

In all experiments, analytical reagent grade chemicals were used, solutions were prepared with ultrapure deionised water (18.2 MU cm, Milli-Q Advantage 10, Millipore) and glassware is acid washed.

To test gel-based BLs under optimized XFM analysis and compare the kapton BL, PU gel-based DGT BLs with Metsorb were prepared. The most conventional binding gels in DGT are poly/or bis-acrylamide gels, however, the polyurethane based gel was previously selected for XFM analysis because of its superior handling and drying for large DGTs and because of its suitability in tandem XFM analysis (Doolette et al. 2022). Polyurethane-based 100 µm-thin binding gels with metsorb were prepared as described in Doolette et al. (2022) according to previously established knife-coating procedures (Kreuzeder et al. 2013). The PU gels were originally developed by Kreuzeder et al. (2013) because its highly stable and tear-proof with superior handling properties compared to polyacrylamide gels. The bis-acrylamide based diffusive layer (spacer thickness 500 µm) was prepared as previously described (Zhang and Davison, 1999). The membrane in contact with the sample is a polyether sulfone (PES) filter membrane (0.45 µm, 60-100 µm thickness, Sterlitech Corporation). The conventional testing of available elements in soil with DGT uses a 0.8 mm thick diffusive gel between the BL and the membrane. In DGT imaging, the diffusive gels are often thin (200 µm) or even omitted and replaced by ultra-thin membranes (10 µm), to limit lateral diffusion in the diffusion layer and therefore reduce image blurring (Santner et al. 2015). To test weather lateral diffusion causes relevant deterioration of the resolution, the DL was omitted in an additional set of Petri dishes with the South-Australia soil and MAP fertilizer for comparison.

Table S1. Properties of the soils collected from South Australia and New South Wales (taken from Arias et al. 2021).

**Soil**	**South Australia**	**New South Wales**
Texture	Clay Loam	Clay
pH $_{1:5\ water}$	8.5	7.4
Organic C (%)	2.1	0.8
Colwell P (mg/kg)	44	17
Olsen P (mg/kg)	11	7
Phosphorus buffering index (PBI)	180	73
$CaCO_3$ equivalent (%)	36	below detection
Iron (mg/kg)	3.5	17
Exchangeable aluminium (cmol (+)/kg)	<0.1	<0.1

3. Kapton binding layer calibration standards

The matrix-matched DGT standards for calibration of the Kapton BLs were prepared by exposing BLs of 7.5 cm^2 to solutions (35 mL) with different P concentrations (0, 0.29, 2.9, 5.7, 14, 29 mg P L^{-1}) in duplicate at pH 6.5 and 10 mM NaCl background on a horizontal shaker. The BLs were exposed for 24 h, thereby yielding different P loadings. Subsections of the exposed BLs were made. Small sections (3 mm height, about 2 mm width, e.g. Figure S4) were used for calibration of the XFM and LA-ICP-MS analysis, whereas the remaining part was eluted for 24h in 5 mL of 0.2M NaOH and P concentrations in eluates were measured by ICP-MS after dilution and 2% HNO_3 acidification (Agilent 8900). Initial test showed that with 1 M NaOH, commonly used to elute P from Metsorb-based BLs (Panther et al. 2010), the tape slightly degraded after 24h. Therefore, 0.2 M NaOH was subsequently used for elution, which yielded the same P elution efficiency than the higher NaOH concentration. The P loadings on the gel derived from elution was confirmed by microwave digestion for two different P loadings (results not shown). The calibration standards for XFM analysis include the blank and 5 different P loadings.

4. Laser Ablation ICP-MS imaging

A nanosecond 193 nm ArF excimer-based laser ablation system (Iridia,Teledyne, CETAC) equipped with the Cobalt ablation chamber was coupled to a Triple-Quadrupole ICP-MS (8900 Agilent) for the analysis of a selection of DGT binding layers and matrix-matched standards. The Cobalt long-pulse module with longer washout was used because of the relatively large spot size used (80 µm). Sample introduction in the ICP-MS was done with the Aerosol Rapid Introduction System (ARIS) directly connected to the ICP torch inlet. Laser ablation of the gels was performed in line scan mode, with settings given in Table S1. The LA-ICP-MS settings were chosen to scan the relatively large areas of the binding layers with a limited amount of spots and run time. The spot size was set at 80 µm. The scan speed was 1600 µm s-1 with a duty cycle of 100 ms, resulting in a pixel size in the scan direction of 160 µm. Horizontal lines were ablated on the gels with vertical spacing of 160 µm to obtain square 160 µm x 160 µm pixels. The number of lines per gel ranged between 275 and 300 and the line length about 40 mm, the total analysis time per gel was 180 – 200 min (excluding the calibration binding layers).

The Kapton binding layers for calibration were prepared as described for the XFM analysis, i.e. following 24 h adsorption in P solutions at pH 6.5, with P surface loading determined from elution. Two higher calibration standards were included compared to XFM analysis. A calibration curve is determined by plotting the P intensity (counts) versus the different P mass loadings(ng/cm^2). The calibration standards were measured at the start and end of the sample run to correct for signal drift. The sensitivity decreased up to a factor two during some runs. Data processing is done with Iolite v4 software (http://www.iolite.org.au; e.g., Hellstrom et al. 2008). The 3D Trace Elements data reduction scheme from Iolite software was used to subtract the gas blank and to correct for sensitivity drift using the slope of the calibration standards measured at the start and end of each run. The average P loadings in the blank (n=2) is low (5.1 ng P cm^{-2}) and therefore no subtraction was made for that P content of the gel blanks.

Table S2 Operational parameters for LA-ICP-MS imaging

**Teledyne CETAC Technologies Iridia Laser-Ablation system**		
Scan mode		Line scanning
Beam size (square)	µm	80
Interline distance	µm	160
Fluence	J cm^{-2}	1.5
Dosage		4
Repetition rate	Hz	40
Scan speed	µm s^{-1}	1600
He carrier gas flow rate	L min^{-1}	0.6
Cobalt cell – long pulse module		
**Agilent 8900 ICP-QQQ**		
Rf power	W	1350
Sampling depth	mm	7.5
Nebuliser gas	L min^{-1}	1
dwell time isotopes	ms	^{13}C:7, ^{31}P:65, ^{47}Ti:20
Duty cycle	ms	100

6. Image processing and statistical analysis

The 2D-maps of surface loadings of P on the BL (ng P cm^{-2}) obtained with XFM analysis were converted to 2D-maps of labile P concentrations (µg P L^{-1}) using the DGT equation (Eqn.S1) (Zhang and Davison, 1994),

$$C_P = \frac{M * \Delta g}{A * D * t} \qquad \text{(Eqn.S1)}$$

where M/A (ng P cm^{-2}) the P surface loading on the BL accumulated during the deployment time, Δg the diffusive layer thickness (cm), D the diffusion coefficient for PO_4 through a membrane-based bis-acrylamide diffusive layer, taken from Arias et al. 2022, and t deployment time (s).

The diffusion coefficients of polyphosphates (present in the APP fertilizer) in the DL were not determined. Noting that diffusion coefficients are usually lower in hydrogels compared to water (Davison & Zhang, 2016), the diffusion coefficient of the pyrophosphate anion in water ($P_2O_7^{4-}$, 6.39 x 10^{-6} cm^2 s^{-1} at 25°C) is lower than that of orthophosphate (PO_4^{3-}, 8.24 x 10^{-6} cm^2 s^{-1}) (Vanysek, 2013), which suggests that labile P concentrations from the APP fertilizer could be underestimated by a maximum of 23% if pyrophosphate was the only species present on the BL. However, this is likely the maximum underestimation as pyrophosphate is the dominant form of P present in APP with orthophosphate and more condensed forms of P present at lower concentrations (McBeath et al. 2007).

The 2D maps of C_P are presented as contour plots plotted using the software program R v4.2.3 for which C_P values < 0 are set at zero (Figure 2). To quantitatively compare P diffusion in both soils and different fertilisers, the C_P concentrations are plotted as function of the radial distance R from fertilizer application. To identify the pixel corresponding to the point of fertilizer application, a five-pixel average of the 2D C_P map was calculated first in ImageJ and the centre of the maximum values was set to the origin of the new radial axis R at value of 0 µm. All other pixels with coordinates x,y are then converted to radial distance from the centre of fertilizer application to make the plot of C_P versus r including all measured pixels (i.e. > 200 000 datapoints depending on scanned area). In the plots of C_P

versus R, the concentration values are averaged in size bins of 150 µm (or 160 µm for LA-ICP-MS) and the 95% confidence interval on each size bin is calculated. The P diffusion profiles measured in the 18 samples generally exhibited a sigmoidal pattern (Figure S6). Therefore, a 4 parameter log-logistic model describing the C_P concentration as function of radial distance was fit on each of the plots and relevant model parameters are statistically compared. These diffusion profiles were not reconstructed by numerical modelling since not all required soil properties are available (Degryse et al. 2014). Instead, the measured P diffusion profiles were empirically fitted with a log-logistic curve with four adjustable parameters to describe P solution distribution according to Eqn. (S2):

$$C_P(r)= C_{P,\,back}+ \frac{C_{P,\,max}- C_{P,\,back}}{1+e^{\left(-a(R-b)\right)}} \qquad \text{(Eqn.S2)}$$

with $C_p(r)$ the C_P (µg P L^{-1}) concentration as function of r(mm), $C_{P,back}$ (µg P L^{-1}) the lower asymptote value corresponding to the concentration far from fertilizer application, $C_{P,max}$ (µg P L^{-1}) the upper asymptote value corresponding with the concentration at fertilizer application, a the slope (mm^{-1}) and b (mm) the distance at which the P concentration is intermediate between $C_{P,back}$ and $C_{P,max}$. The profiles of all 18 were fitted with the nonlinear fitting routine in the statistical program JMP (JMP®, Version 17. SAS Institute Inc., Cary, NC, 1989–2023) using the Newton steepest descent algorithm based on the minimization of the sum of squared errors between observed and modelled C_P values.

The radius of P diffusion (R_{diff}) is ideally determined as the signal that differs from a soil sample without fertilizer application, i.e. the P background concentration needs to be known, but this was not analyzed. The low $C_{P,back}$ values obtained from Eqn.2 (not shown) suggests that the P background concentration is likely below the detection limit (LOD) for both soils. Therefore, the R_{diff} is arbitrary defined here as the distance from fertilizer application where C_P decreases below the LOQ (10 times the standard deviation in blanks, 24 µg P L^{-1}) determined from the XFM calibration curve and converted to labile P concentrations with Eqn. 1. The differences in parameter values $C_{P,max}$ and R_{diff} among different fertilizers of the same soil or among soils are compared statistically within soil using

the student t-test at significance level alfa 0.05 using individual petri dishes as sampling replicates (3) for each fertilizer and soil.

To compare XFM versus LA-ICP-MS analysis, P surface loadings (M_P) on the binding layer measured with both methods are plotted as function of the radial distance R from fertilizer application and the parameters $M_{P,max}$ and R_{diff} are compared statistically from the log-logistic fit analogously to Eqn. S2 but with surface loadings instead of P DGT concentrations:

$$M_P(r) = M_{P,\ back} + \frac{M_{P,\ max} - M_{P,\ back}}{1+e^{\left(-a(R-b)\right)}} \qquad \text{(Eqn. S3)}$$

the R_{diff} is the distance from fertilizer application where M_P decreases below the LOQ (10 times the standard deviation in blanks, 100 ng P cm^{-2}) determined from the XFM calibration curve.

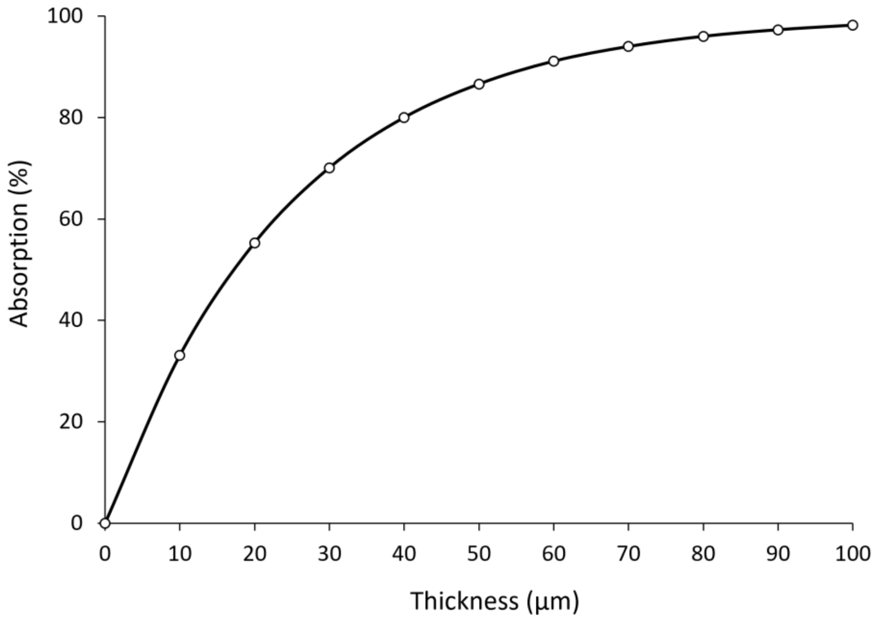

Figure S1 Simulation of absorption of P fluorescence signal (energy 2.1 keV) as function of thickness of polyurethane gel overlaying a (Metsorb) surface containing P. The calculations show that even a 10 µm thick gel layer absorbs already almost 40 % of the fluorescence signal. Calculations are made with the Xray Utils database (Xray Utils (quantumdetectors.github.io)).

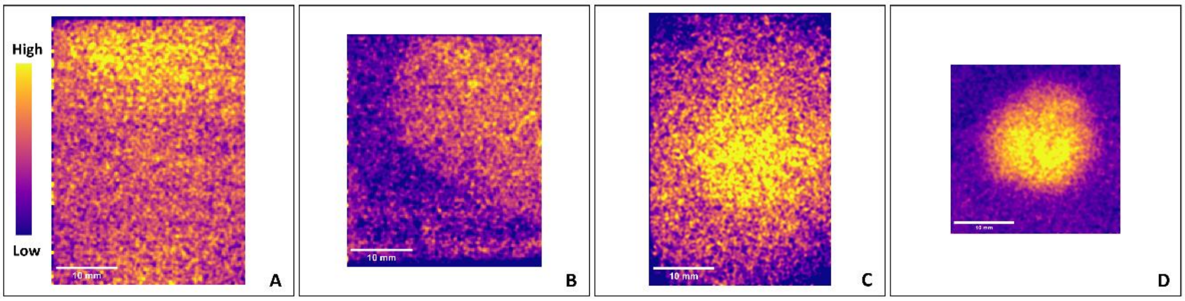

Figure S2 The 2D maps of P from XFM analysis showing polyurethane gels applied on the New South Wales soil with MAP fertilizer (2 replicates panel A and B) and corresponding 2D map of P on the kapton binding layer (panel C) and 2D map of P on the Kapton binding layer from the South-Australia soil with MAP fertilizer (panel D). The P distribution is almost random in the PU gels and differs clearly between replicates whereas the P distribution is as expected from P diffusion experiment on the Kapton binding layers.

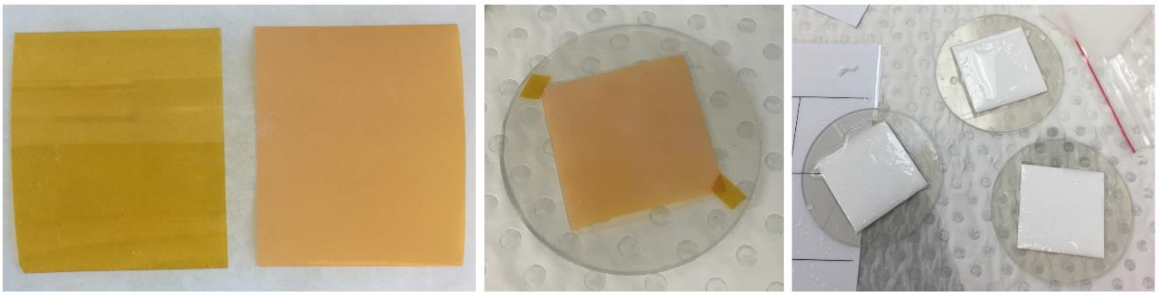

Figure S3 Kapton tape before (left) and after (right) addition of Metsorb showing the homogeneous application of Metsorb on the tape. The picture in the middle shows the Kapton binding layer in the 3D-printed (Vero Clear resin) support stuck using two pieces of tape in the corners, and the right picture shows the full DGT assembly including the PES 0.45 µm membrane, followed by (not visible) diffusive layer and Kapton binding layer before DGT deployment.

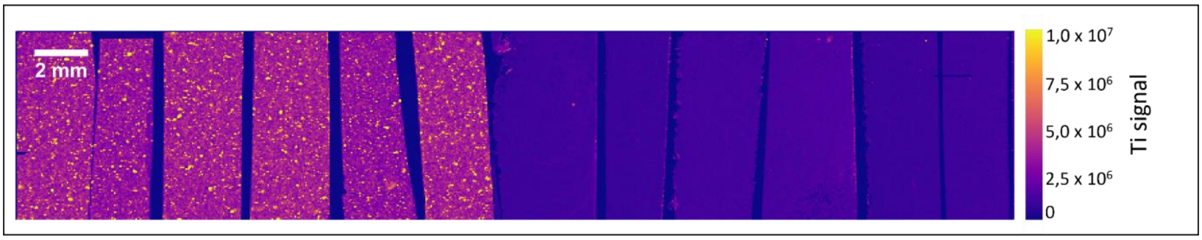

Figure S4 Titanium (Ti) intensity signal measured with XFM to determine the distribution of the Ti-based binding agent Metsorb in/on the DGT binding layers. The series of P calibration standards are shown, the first 6 are the gel-based PU binding layers with Metsorb, the next 6 are the Kapton binding layers with Metsorb. The average Ti signal is about 4 times higher for the PU binding gels, i.e. 3.8×10^6 (standard error 2.5×10^4) than for the kapton binding layers 1.0×10^6 (standard error 1.9×10^3), but more heterogeneous given the higher standard error. The reduction in signal for Ti due to self-absorption could be up to 27% for a 100 µm thick PU gel.

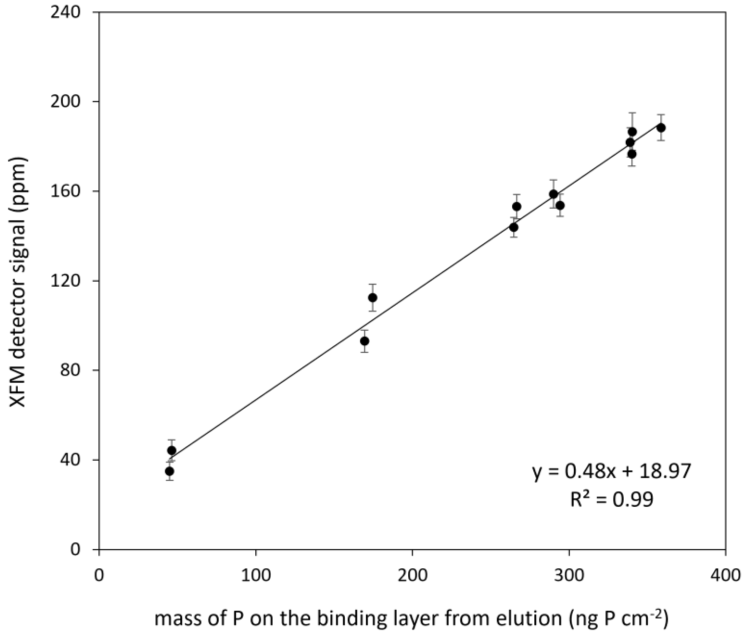

Figure S5 Calibration curve to convert the XFM fluorescence signal (ppm) to P mass loadings (ng P cm^{-2}) on the Kapton binding layer using on matrix-matched calibration standards. The bars are standard errors.

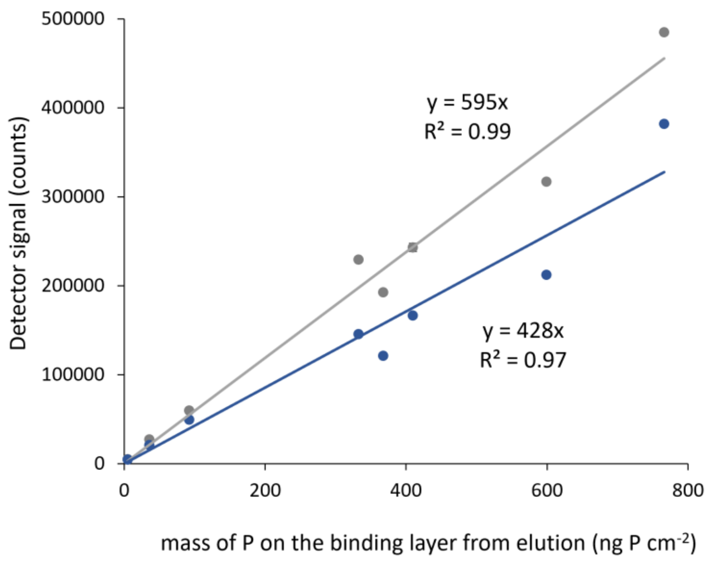

Figure S6 Calibration curve to calibrate the LA-ICP-MS analysis using matrix-matched Kapton calibration standards. The bars showing standard errors are indicated but are very small. The highest sensitivity was obtained at the start of the analysis (grey), but the sensitivity decreased towards the end of the run (blue). The decrease in sensitivity is taken into account using the slopes assuming a linear decrease in sensitivity during the analysis.

13. Diffusive layer thickness

Figure S7 shows the effect of using a diffusive gel on the accumulated mass and P distribution on the BL. The standard DGT deployment with a diffusive layer (diffusive gel + protective membrane) did not show any widening of the P distribution profiles for the spatial resolution of the measurements compared to using the membrane (100 µm) alone as the DL. However, the accumulated mass of P is about 2 times higher when using the membrane only, hence, the calculated DGT concentrations would be 4 times smaller for the 8 times thinner diffusion layer because the DGT concentration is a linear function of the diffusion layer thickness (Eqn. S1). Pore water concentrations obtained with imaging DGT with thin diffusive layers are mostly unrealistically small. Therefore, DGT images obtained with thin DLs are mostly presented as fluxes, or as surface loadings on the BL instead of DGT concentrations (Santner et al. 2015; Wagner et al. 2020). We preferred here to use diffusive layers with thickness similar as in conventional DGT samplers to obtain DGT concentrations comparable to values measured with bulk samplers.

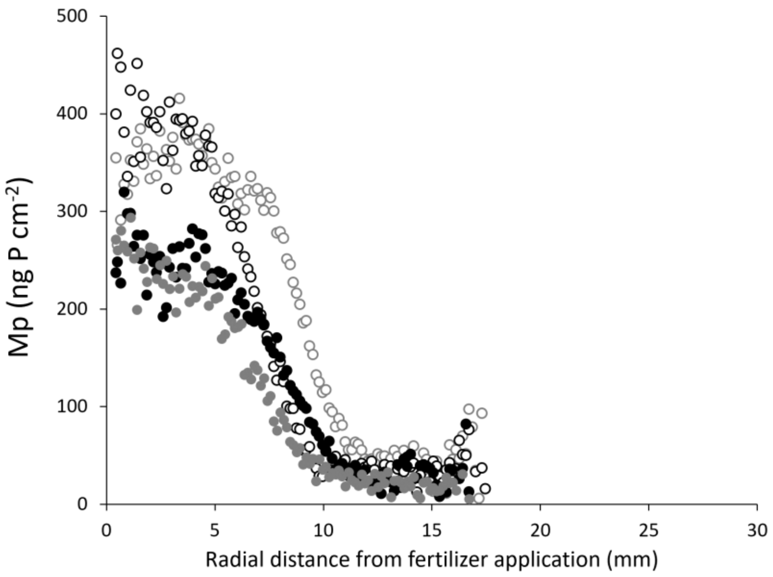

Figure S7 XFM analysis of the P loading M_p (ng P cm^{-2}) on Kapton BLs obtained from DGT deployment with (filled dots) and without diffusive layer (unfilled dots) for the South-Australia (SA) soil with MAP fertilizer.

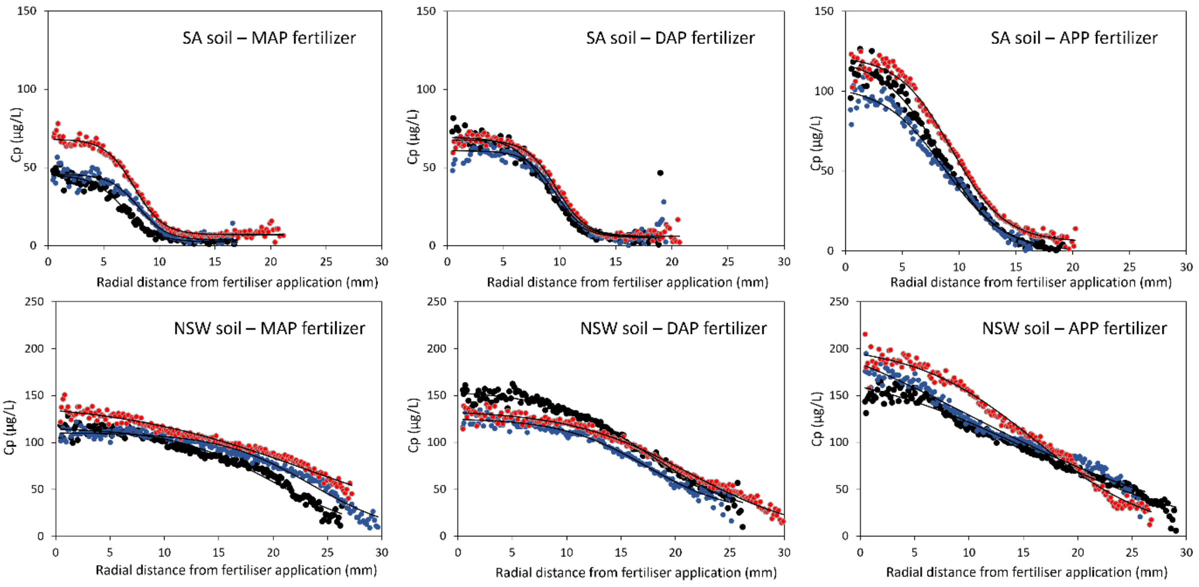

Figure S8 Individual replicates of the Kapton binding layers (n=3) analyzed by XFM. The DGT concentration C_p (µg P L^{-1}) as function of radial distance of fertilizer application are derived from the 2D diffusion profiles in the South Australia soil (top) and New South Wales soil (bottom) with MAP, DAP and APP fertilizer. The lines indicate the log-logistic model fit.

16. References

Arias DM, Teasdale PR, Doolette CL, Lombi E, Farquhar S, Huang J. Chemosphere. **2021**. 269:128704

Davison, W., Zhang, H. **1994***. Nature* 367, 546–548

Davison W., Zhang H. (2016). Principles of measurements in simple solutions, in Diffusive Gradients in Thin-Films for Environmental Measurements, ed Davison W. (Cambridge: Cambridge University Press;), 24–27. 10.1017/CBO9781316442654.003

Degryse, F. and McLaughlin, M.J. **2014**. Soil Science Society of America Journal, 78. 832-842

Doolette CL, Howard DL, Afshar N, Kewish CM, Paterson DJ, Huang J, Wagner S, Santner J, Wenzel WW, Raimondo T, De Vries Van Leeuwen AT, Hou L, van der Bom F, Weng H, Kopittke PM, Lombi E. J.Anal.Chem. **2022**. 22;94(11):4584-4593

Hellstrom, John & Paton, Chad & Woodhead, Jon & Hergt, Janet. **2008**. Mineralogical Association of Canada short course series. 40. 343–348

Kreuzeder, A., Santner, J., Prohaska, T., Walter W. Wenzel. *Analytical Chemistry* **2013** *85* (24), 12028-12036

McBeath, T.M., Lombi, E., McLaughlin, M.J. and Bünemann, E.K. (2007), Polyphosphate-fertilizer solution stability with time, temperature, and pH. Z. Pflanzenernähr. Bodenk., 170: 387-391. https://doi.org/10.1002/jpln.200625166

Panther, J.G, Teasdale, P.R., Bennett, W.W., Welsh, D.T., Zhao,H., Environ. Sci.Technol. **2010.** *44* (24), 9419-9424

Santner, J.; Larsen, M.; Kreuzeder, A.; Glud, R. N. *Analytica Chimica Acta.* **2015**, *878*, 9–42

Zhang, H.; Davison, W. Anal. Chim. Acta. **1999,** 398, 329−340

Vanysek, P., 2012-2013. Ionic Conductivity and Diffusion at Infinite Dilution, in: Rumble, J.R. (Ed.), Handbook of Chemistry and Physics. 93RD edition.Taylor & Francis Ltd, p. 1604.

Wagner S., Hoefer C., Prohaska T., Santner J. **2020**, Two-Dimensional Visualization and Quantification of Labile, Inorganic Plant Nutrients and Contaminants in Soil. J Vis Exp. Sep 1;(163)
